# SuperHistopath: A Deep Learning Pipeline for Mapping Tumor Heterogeneity on Low-Resolution Whole-Slide Digital Histopathology Images

**DOI:** 10.3389/fonc.2020.586292

**Published:** 2021-01-20

**Authors:** Konstantinos Zormpas-Petridis, Rosa Noguera, Daniela Kolarevic Ivankovic, Ioannis Roxanis, Yann Jamin, Yinyin Yuan

**Affiliations:** ^1^ Division of Radiotherapy and Imaging, The Institute of Cancer Research, London, United Kingdom; ^2^ Department of Pathology, Medical School, University of Valencia-INCLIVA Biomedical Health Research Institute, Valencia, Spain; ^3^ Low Prevalence Tumors, Centro de Investigación Biomédica en Red de Cáncer (CIBERONC), Instituto de Salud Carlos III, Madrid, Spain; ^4^ The Royal Marsden NHS Foundation Trust, London, United Kingdom; ^5^ Breast Cancer Now Toby Robins Research Centre, The Institute of Cancer Research, London, United Kingdom; ^6^ Division of Molecular Pathology, The Institute of Cancer Research, London, United Kingdom

**Keywords:** deep learning, machine learning, digital pathology, computational pathology, tumor region classification, melanoma, neuroblastoma, breast cancer

## Abstract

High computational cost associated with digital pathology image analysis approaches is a challenge towards their translation in routine pathology clinic. Here, we propose a computationally efficient framework (SuperHistopath), designed to map global context features reflecting the rich tumor morphological heterogeneity. SuperHistopath efficiently combines i) a segmentation approach using the linear iterative clustering (SLIC) superpixels algorithm applied directly on the whole-slide images at low resolution (5x magnification) to adhere to region boundaries and form homogeneous spatial units at tissue-level, followed by ii) classification of superpixels using a convolution neural network (CNN). To demonstrate how versatile SuperHistopath was in accomplishing histopathology tasks, we classified tumor tissue, stroma, necrosis, lymphocytes clusters, differentiating regions, fat, hemorrhage and normal tissue, in 127 melanomas, 23 triple-negative breast cancers, and 73 samples from transgenic mouse models of high-risk childhood neuroblastoma with high accuracy (98.8%, 93.1% and 98.3% respectively). Furthermore, SuperHistopath enabled discovery of significant differences in tumor phenotype of neuroblastoma mouse models emulating genomic variants of high-risk disease, and stratification of melanoma patients (high ratio of lymphocyte-to-tumor superpixels (p = 0.015) and low stroma-to-tumor ratio (p = 0.028) were associated with a favorable prognosis). Finally, SuperHistopath is efficient for annotation of ground-truth datasets (as there is no need of boundary delineation), training and application (~5 min for classifying a whole-slide image and as low as ~30 min for network training). These attributes make SuperHistopath particularly attractive for research in rich datasets and could also facilitate its adoption in the clinic to accelerate pathologist workflow with the quantification of phenotypes, predictive/prognosis markers.

## Introduction

The analysis of histopathological images of surgical tissue specimens stained with hematoxylin and eosin (H&E) remains a critical decision-making tool used for the routine management of patients with cancer and the evaluation of new therapeutic strategies in clinical trials (1–3). In several precision medicine settings, there is an increasing demand for accurate quantification of histological features. However, in their diagnostic practice, pathologists exercise a predominantly qualitative or semi-quantitative assessment with an inherent degree of inter- and intra-observer variability, which occasionally hampers their consistency (4–7). In the new era of digital pathology, advanced computational image analysis techniques are revolutionizing the field of histopathology by providing objective, robust and reproducible quantification of tumor components, thereby assisting pathologists in tasks such as tumor identification and tumor grading (8, 9). Histopathological image analysis can now be performed in high-resolution H&E-stained whole-slide images (WSI) using state-of-the-art deep learning and classical machine learning approaches for single cell segmentation and/or classification. The new ability to map the spatial context of each single cell also opened new avenues for the study of the tumor micro-environment (10–16), which is key to guide the delivery of precision medicine including immunotherapy.

However, computational pathology is still not widely adopted in the oncological setting. One of the challenges lies in the gigabyte sizes of high-resolution WSIs, which result in computationally expensive approaches. WSIs need to be divided into images patches (typical size: 256x256) before being processed by deep networks such as convolutional neural networks (CNNs) (17). Secondly, single-cell approaches provide markers that are often hard-to-be-evaluated or even interpreted by the pathologists and can be prone to the generalization errors when applied in new unseen dataset. As a result, many promising markers eventually fail to reach the clinic due to a lack of cross-validation in new independent datasets. On the other hand, tissue classification approaches, which target multicellular assemblies and paucicellular areas where individual cells are incorporated into the region segmentation, would be accessible for visual validation by pathologists. Such algorithms would enable the characterization of the distribution and interrelationship of global features that are currently detectable by human perception but not quantifiable without artificial intelligence- (AI-)assisted numerical expression.

Current computed pathology tools primarily focus on individual cell analysis at high-resolution (40x/20x magnification) with limited local context features, whereas pathologists frequently employ collateral information, taking into account the overall tissue microarchitecture. Many established clinical markers are actually identified at low or intermediate magnifications, including tumor architecture-based grading systems (18, 19), stroma-tumor ratio (20, 21), infiltrating lymphocytes (TILs) (22, 23) and necrosis (24–26). This has not been yet fully emulated by computational pathology methodologies. However, some methods for the classification of tissue components have been suggested either using image patch classification typically with a CNN or pixel-level classification/segmentation typically with a U-Net-like architecture (27), mainly for tasks such as the dichotomized classification of tissue (e.g. cancerous vs non-cancerous) (28, 29), the segmentation of a feature of interest (e.g. glands) (16, 30) or multi-type tissue classification (9, 31–35). For segmentation purposes, U-Net-like architectures are usually preferred over CNNs, which have established limitations in conforming to object contours. Yet, CNNs have also resulted in promising segmentation approaches (36–38) with the enhanced capability of classifying a large number of categories (39). Multi-scale approaches incorporating information from various image resolutions have also been proposed (40–43). Different approaches have been explored for the classification of epithelium or stroma using superpixels-based segmentation of image patches with either hand-crafted or deep learning features (44, 45). Bejnordi and colleagues used a similar method for their multi-scale approach for the classification of tissue or non-tissue components on low resolution images and stroma and background regions from intermediate and high resolution images (46). However, these methods are typically performed on high-magnifications image patches (20-40x and more rarely 10x) and are associated with a high computational cost.

Here, we propose a framework (SuperHistopath), which can map most of the global context features that contribute to the rich tumor morphological heterogeneity visible to pathologists at low resolution and used for clinical decision making in a computationally efficient manner. We first apply the well-established simple linear iterative clustering (SLIC) superpixels algorithm (47) directly on the WSI at low resolution (5x magnification) and subsequently classify the superpixels into different tumor region categories using a CNN based on pathologists’ annotations. SuperHistopath particularly capitalizes on:

- the use of superpixels which provide visually homogeneous areas of similar size respecting the region boundaries and avoid the potential degradation of classification performance associated with image patches, (no matter how small) spanning over multiple tissue categories.
- the use of CNN necessary to accurately classify and map the multiple tissue categories that constitute the rich and complex histological intratumoral heterogeneity.
- the computational efficiency, faster processing speed and lower memory requirements associated with processing the WSI at low resolution.

We applied SuperHistopath to H&E-stained images from three different cancer types: clinical cutaneous melanoma, triple-negative breast cancer and tumors arising in genetically-engineered mouse models of high-risk childhood neuroblastoma.

## Materials and Methods

### Datasets

All digitized whole-slide images (WSI) used in this study were H&E-stained, formalin-fixed and paraffin-embedded (FFPE) sections, and scaled to 5x magnification as presented in **Table 1** (image sizes at 5x varied from ~8000x8000 to ~12000x12000 pixels). We applied our framework to clinical patient samples of cutaneous melanoma and triple-negative breast cancer, in addition to tumor samples from transgenic mouse models of childhood neuroblastoma. Both the Th-*MYCN* and Th-*ALK^F1174L^/MYCN* mouse models have been shown to spontaneously develop abdominal tumors, which mirror the major histopathological characteristics of childhood high-risk disease (50, 51).

**Table 1:** Summary of the datasets used.

Cancer type	Number of WSIs	Digital scanner	Pixel resolution (5x magnification)	Dataset
Cutaneous melanoma	127	Aperio ImageScope	2.016 μm	The Cancer Genome Atlas (TCGA)
Triple-negative breast cancer	23	NanoZoomer XR	2.3 μm	Internal dataset, Collaboration with The Serbian Institute of Oncology
High-risk neuroblastoma (mouse models)	73	NanoZoomer XR	2.3 μm	Internal dataset Tumors samples coming from established Th-*MYCN* and Th-*ALK^F1174L^/MYCN* transgenic mouse colonies (48, 49) and processed by a clinical histopathological core facility

### Region Classification

**Figure 1:**
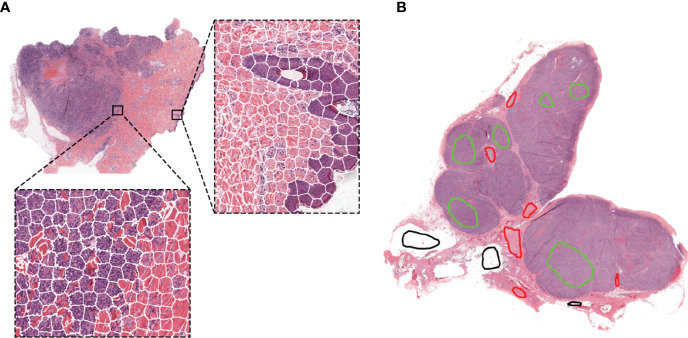
Representative examples of the SLIC superpixels segmentation and ground-truth annotations in TCGA melanoma samples **(A)** Whole-slide image segmentation using the SLIC superpixels algorithm. Note how the superpixels adhere to the boundaries of the different components of the tumor with each superpixel containing a single type of tissue **(B)** Ground-truth annotations are provided by the pathologists by marking samples of the region components (the different colors represent different regions) without the need for delineating the boundaries of the tumor components.

First, each dataset was pre-processed using the Reinhard stain normalization (52) to account for stain variabilities that could affect classification. Then, all images were segmented using the simple linear iterative clustering (SLIC) superpixels algorithm, which groups together similar neighboring pixels. With our pathologist’s input, we selected the optimal number of superpixels by visually identifying a superpixel size that capture only homogeneous areas and adhere to image boundaries. This is a critical step for ensuring accurate tissue segmentation, and therefore, classification (**Figure 1**). The number of superpixels was adapted for each image to ensure a homogenous superpixel size across the datasets and was automatically set based on the image size according to *Equation 1* (53).

(1) $$N_{i}=ceiling\left(\frac{S_{i}}{U}\right)$$

where *Ni* is the number of superpixels in the *i^th^* image, *Si* is the size of image *i* in pixels, and *U* is a constant held across all images that defined the desired superpixels size.

The SLIC algorithm inherently provides a roughly uniform superpixel size. Setting *U* = 1500, *Equation 1* gave a mean superpixels size of 51 × 51 pixels, equivalent to an area of approximately 117 × 117 μm^2^. Bilinear interpolation was subsequently use to resize each superpixel to a fixed size of 56 x 56 or 75 x 75 pixels (the minimum input size for inception-like network architectures).

Region annotations were provided by a senior pathologist with over 20 years of experience for the melanoma and breast cancer clinical datasets, and a senior pediatric neuropathologist with over 20 years of experience for the neuroblastoma mouse datasets. For training and testing, superpixels were assigned to each category based on their isocenter location within the annotated regions. Note that region annotations for our algorithm do not need to delineate boundaries as illustrated in **Figure 1B**.

The numbers of clinically relevant tissue categories, number of WSIs and superpixels used for training and testing are summarized for each tumor types in **Table 2**. Standard image augmentations, such as rotations (90°, -90°, 180°), flips (horizontal and vertical), and contrast (histogram equalization) were performed in each case to capture more variation and even out the training dataset imbalances.

**Table 2:** Summary of the datasets used for training and testing the convolutional neural network.

Cancer type	Number of WSIs used for network training		Regional classification	
Cutaneous melanoma	Total	27	***6 categories***	***Superpixels for training***
	Training	22	Tumor tissue	21940
	Testing	5	Stroma	12419
			Normal epidermis	1646
			Lymphocytes cluster	2367
			Fat	15484
			Empty/white space	3412
Triple-negative breast cancer	Total	23	***6 categories***	***Superpixels for training***
	Training	18	Tumor tissue	18873
	Testing	5	Stroma	24220
			Necrosis	15102
			Lymphocytes cluster	3472
			Fat	10044
			Empty/white space	16473
High-risk neuroblastoma (mouse model)	Total	60	***8 categories***	***Superpixels for training***
	Training	44	Region of undifferentiated neuroblasts	20512
	Testing	16	Tissue damage (necrosis/apoptosis)	17645
			Differentiation region	5740
			Lymphocytes cluster	4009
			Hemorrhage (blood)	6124
			Muscle	6415
			Kidney	14976
			Empty/white space	21470

Note that the testing datasets consisted of whole-slide images from different patients from the training dataset.

### Training of the Convolutional Neural Networks

**Figure 2:**
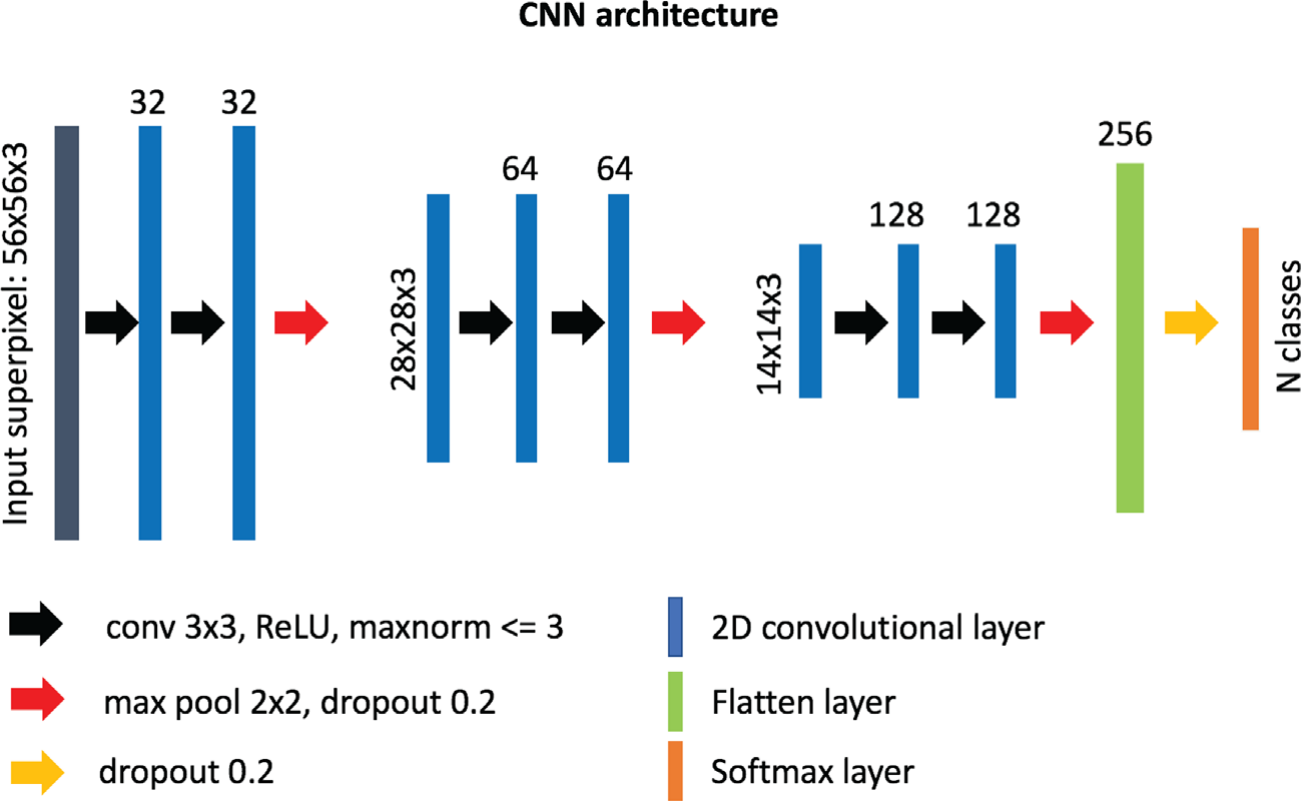
Architecture of our custom-designed convolutional neural network for the classification of superpixels into different tissue-level categories.

Our custom-designed CNN for superpixel classification consists of 6 convolutional layers (32, 32, 64, 64, 128, 128 neurons, respectively) of 3 x 3 filter size and 3 max-pooling layers, followed by a “flatten” layer and a dense layer of 256 neurons (**Figure 2**). A superpixel RGB image (post-interpolation) was used as input into the network and normalized from range 0–255 to range 0–1 using the maximum value. The output of the network was a label assigned to each superpixel based on which region category it belonged to. After empirical experimentation, a ReLU activation function was used in all layers except for the last layer where standard softmax was used for classification. The weights incident to each hidden unit were constrained to have a norm value less than or equal to 3 and a dropout unit of 0.2 was used before every max-pooling operation to avoid overfitting (54). The weights of the layers were randomly initialized using “Glorot uniform” initialization (55), and the network was optimized using the Adam method (56) with a learning rate of 10^-3^ and a categorical cross-entropy cost function. The number of trainable parameters for our custom-made network is ~1.9 M. The network was implemented in python (v. 3.6.5) using the Keras/Tensorflow libraries (v. 2.2.4/1.12.0, respectively).

To choose the best network for our framework, we tested other known neural network architectures as implemented in the Keras framework, including InceptionV3 (57), Xception (58), InceptionResNetV2 (59), and ResNet (60). We initialized the weights using the pre-trained ImageNet weights. To optimize each network, we excluded the final classification layer, and added three additional layers, i) a global average pooling layer, ii) a dense layer of 256 neurons with ReLu activation, constrained to have a norm value less than or equal to 3, and iii) a dense layer tailored to the number of classes of each cancer type using the softmax function for classification.

For inception-like architectures (Inception v3, InceptionResNetV2, Xception) only superpixels of size 75 x 75 were used. We trained all the networks for 50 epochs using batch sizes of 150 and 256 for superpixels of sizes 75 x 75 and 56 x 56, respectively, and kept the models with the highest validation accuracy.

The Xception and custom-made networks were re-trained from the beginning for each cancer type, without applying any further changes.

### Application of SuperHistopath for the Quantification of Clinical Features of Interest

In the melanoma dataset, we calculated the number of pixels belonging to each classified category. For each patient we derived i) the ratio of pixels classified as stroma region to all pixels in tumor compartments, and ii) the ratio of pixels classified as clusters of lymphocytes to all pixels in tumor compartments; we evaluated the prognostic value of these quantitative indices using survival analysis. Patients were divided into high- and low-risk groups based on split at the median value of all scores to ensure both groups were of similar size. Kaplan-Meier estimation was used to compare overall survival in the 127 patients. Differences between survival estimates were assessed with the log-rank test and hazard ratios were calculated using Cox’s proportional-hazard regression.

In the neuroblastoma dataset, we evaluated the differences in phenotype between the Th-*ALK^F1174L^*/*MYCN* (n=7) and Th-*MYCN* tumors (n=6) by quantifying the proportion of pixels classified by our SuperHistopath as regions rich in undifferentiated neuroblasts, differentiating neuroblasts, tissue damage (necrosis/apoptosis) hemorrhage and clusters of lymphocytes. Note that i) we did not quantify stroma in these tumors as they faithfully mirror the stroma-poor phenotype which define high-risk disease ii) lymphocytes clusters universally correspond to encapsulation of lymph node by the tumor, rather that tumor infiltrates, consistent with the “cold” immune phenotype of high-risk disease. We focus on identifying any significant difference in the ratio of differentiation or the ratio of hemorrhagic regions to all tumor compartments between the two tumor types using the Mann-Whitney U test, with a 5% level of significance.

## Results

### SuperHistopath Can Accurately Map the Complex Histological Heterogeneity of Tumors

#### Melanoma

We first developed and evaluated our framework on the H&E-stained, FFPE sections of clinical specimen of cutaneous melanoma scaled to 5x magnification. **Figure 1** shows the results of the segmentation using the simple linear iterative clustering (SLIC) superpixels algorithm, which groups together similar neighboring pixels.

**Figure 3:**
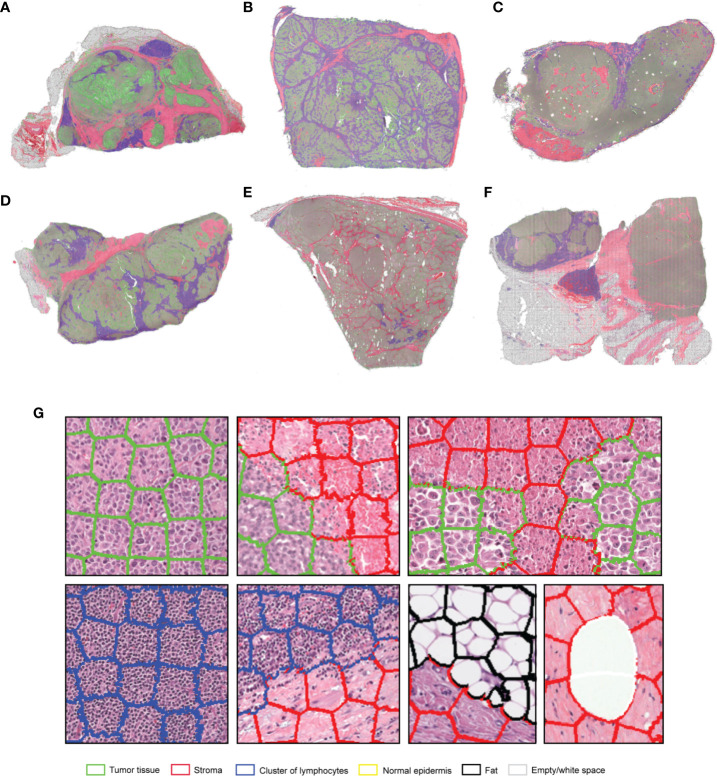
**(A–F)** Representative examples of the results obtained from the application of the SuperHistopath pipeline in whole-slide images of tumors (5x) of the Cancer Genome Atlas (TCGA) melanoma dataset [**(G)** Magnified regions of interest]. Note the important clinically-relevant phenotypes characterized by clusters of lymphocytes infiltrating the tumor in samples **(B, D)**. or the majority of clusters of lymphocytes residing just outside the tumor area (left and central part) with only a few clusters infiltrating the tumor (right part) in sample **(C)**.

The optimized Xception network achieved the highest score and classified the melanoma sample regions into 6 predefined tissue categories of interest: tumor tissue, stroma, cluster of lymphocytes, normal epidermis, fat, and empty/white space with an overall accuracy of 98.8%, an average precision of 96.9%, and an average recall of 98.5% over 14,092 superpixels in a separate test set of five images (**Tables 3**, **4**). Our custom CNN also achieved comparable performance to the state-of-the-art networks with an overall accuracy of 96.7%, an average precision of 93.6%, and an average recall of 93.6% (**Figure 2**, **Supplementary Table 1**). The confusion matrices for the XCeption and our custom CNN networks are presented in **Table 4** and **Supplementary Table 1**, respectively. **Figure 3** shows qualitative results of our approach’s regional classification in representative melanoma WSIs using the optimized Xception network.

**Table 3:** Evaluation metrics of the different neural network architectures in the TCGA melanoma test dataset.

Network	Accuracy (%)	Precision (%)	Recall (%)	Parameters (in millions)
**InceptionV3**	97.5	94.2	96.7	~22.4
**InceptionResNetV2**	97.7	94.1	97.3	~54.8
**ResNet50**	93.8	92.2	88.9	~24.2
**Xception**	**98.8**	**96.9**	**98.5**	~21.4
**Our custom-made CNN**	96.7	93.6	93.6	**~1.9**

The bold values in the Accuracy (%), Precision (%) and Recall (%) fields indicate the highest value i.e. the best performance achieved amongst the networks under comparison. The bold value in the Parameters (in millions) field indicate the network with the fewer parameters used amongst the networks under comparison.

**Table 4:** Confusion matrix of the classification of superpixels using the optimized Xception network in melanoma patients in 6 categories: tumor, stroma, normal epidermis, cluster of lymphocytes (Lym), fat and empty/white space (separate test set of 5 whole-slide images).

	Tumor	Stroma	Epidermis	Lym	Fat	Empty space
**Tumor**	**5286**	10	7	8	0	0
**Stroma**	9	**986**	0	0	2	0
**Epidermis**	22	0	**545**	0	1	0
**Lym**	0	0	1	**821**	0	0
**Fat**	0	9	0	0	**5603**	3
**Empty space**	0	0	0	0	98	**681**

Overall accuracy = 98.8%, average precision = 96.9%, average recall = 98.5%. The bold values indicate the correct predictions of the network.

#### Breast Cancer

**Figure 4:**
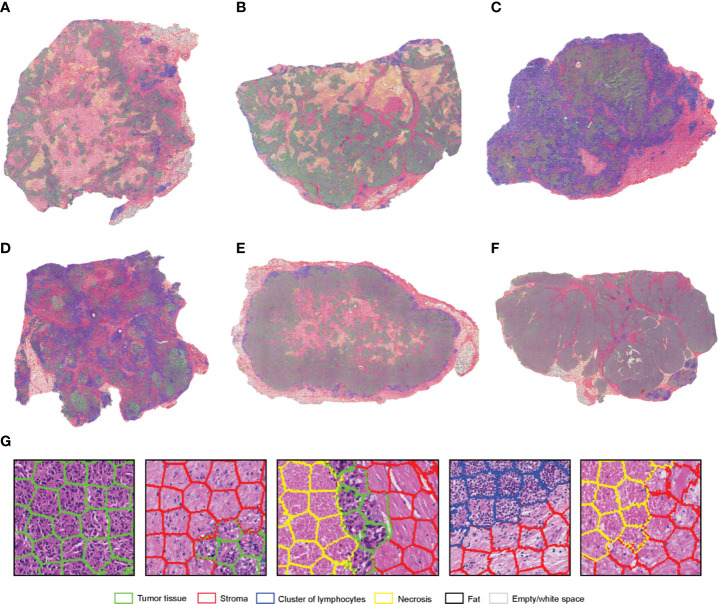
**(A–F)**. Representative examples of the results obtained from the application of the SuperHistopath pipeline in whole-slide images of tumors (5x) of the triple-negative breast cancer **(G)** Magnified regions of interest. Note the important clinically-relevant features, such as the amount of tumor necrosis inside tumors **(A)** and **(B)**, lymphocytes which, are infiltrating the tumor in large number in samples **(C, D)**, but are surrounding the stroma barrier without infiltrating the tumor in samples **(A, B, E, F)**.

SuperHistopath classified sample regions into 6 predefined tissue categories of interest: tumor, necrosis, stroma, cluster of lymphocytes, fat, and lumen/empty space with an overall accuracy of 93.1%, an average precision of 93.9%, and an average recall of 93.6% using Xception and 91.7%, 92.5%, 91.8% respectively using our custom-made CNN over 10,349 superpixels in the independent test set of five images. The confusion matrices for the XCeption and our custom CNN networks are presented in **Table 5** and **Supplementary Table 2**, respectively. **Figure 4** shows qualitative results our approach’s regional classification in representative triple-negative breast cancer WSIs.

**Table 5:** Confusion matrix of the classification of superpixels using the optimized Xception network in triple-negative breast cancer patients in six categories: tumor, necrosis, cluster of lymphocytes (Lym), stroma, fat, and lumen/empty space (separate test set of five whole-slide images).

	Tumor	Necrosis	Lym	Stroma	Fat	Empty space
**Tumor**	**1830**	13	15	42	0	0
**Necrosis**	50	**1446**	2	320	0	0
**Lym**	4	2	**705**	10	0	0
**Stroma**	42	120	20	**3836**	0	1
**Fat**	0	0	0	0	**562**	5
**Empty space**	0	0	0	0	67	**1257**

Overall accuracy = 93.1%, average precision = 93.9%, average recall = 93.6%. The bold values indicate the correct predictions of the network.

#### Neuroblastoma

**Figure 5:**
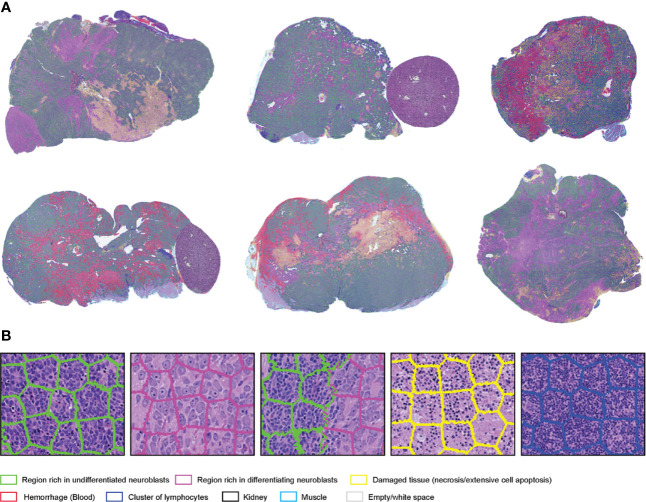
**(A)** Representative examples of the results obtained from the application of the SuperHistopath pipeline in whole-slide images of tumors (5x) arising in genetically-engineered mouse models of high-risk neuroblastoma [**(B)** Magnified region of interest].

SuperHistopath classified the tumor regions into eight predefined tissue categories of interest: undifferentiated neuroblasts, tissue damage (necrosis/apoptosis), areas of differentiation, cluster of lymphocytes, hemorrhage, muscle, kidney, and empty/white space with an overall accuracy of 98.3%, an average precision of 98.5%, and an average recall of 98.4% using Xception and 96.8%, 97.1%, 97.2% respectively using our custom-made CNN over 9,868 superpixels in the independent test set of 16 images. The confusion matrices for the XCeption and our custom CNN networks are presented in **Table 6** and **Supplementary Table 3**, respectively. **Figure 5** shows qualitative results of our approach’s regional classification in representative WSIs of neuroblastoma arising in the Th-*MYCN* mouse model.

**Table 6:** Confusion matrix of the classification of superpixels using the optimized Xception network in the Th-*MYCN* and *Th-*ALK^F1174L^ */*MYCN mouse models in eight categories: region of undifferentiated neuroblasts, necrosis, cluster of lymphocytes (Lym), hemorrhage (blood), empty/white space, muscle tissue and kidney (separate test set of 16 whole-slide images).

	Undifferentiated region	Necrosis	Lym	Differentiation	Blood	Empty space	Muscle	Kidney
**Undifferentiated region**	**1403**	3	0	14	1	0	0	0
**Necrosis**	13	**1642**	1	26	49	2	5	18
**Lym**	6	5	**1150**	0	0	0	0	3
**Differentiation**	0	0	0	**1261**	0	0	0	0
**Blood**	1	7	0	0	**1327**	0	9	0
**Empty space**	0	2	0	0	0	**560**	3	2
**Muscle**	0	2	0	0	1	0	**1176**	0
**Kidney**	0	0	0	0	0	0	0	**1176**

Overall accuracy = 98.3%, average precision = 98.5%, average recall = 98.4%. The bold values indicate the correct predictions of the network.

### SuperHistopath Pipeline for the Analysis of Low-Resolution WSI Affords Significant Speed Advantages

The average time for the SLIC superpixels algorithm to segment a WSI in 5x magnification was < 2 min using a 3.5 GHz Intel core i7 processor. The average time for both the Xception and our custom-made CNN network to classify every superpixel in the images was 1–2 min using the same processor. A quick convergence of the networks (around epoch 30) was observed in all cases, which needed ~3 h for Xception and only ~30 min for our custom-made CNN using a Tesla P100-PCIE-16GB GPU card, and therefore the latter was used for experimenting.

### SuperHistopath Can Provide Robust Quantification of Clinically Relevant Features

#### Stroma-to-Tumor Ratio and Clusters of Lymphocytes Abundance as Predictive Markers of Survival in Melanoma

**Figure 6:**
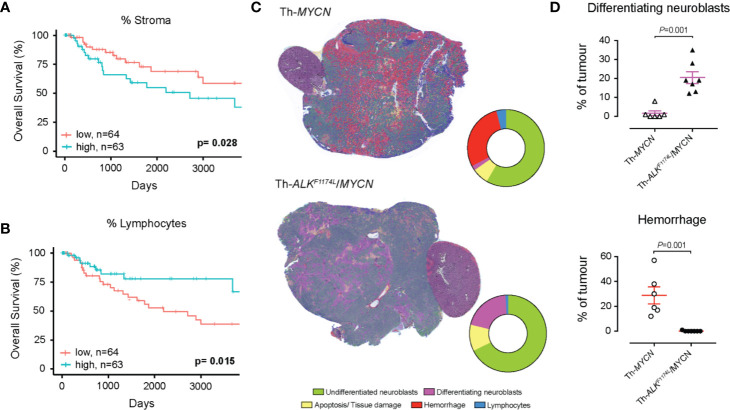
Quantification of clinically relevant features with SuperHistopath. **(A, B)** show associations between survival outcomes and SuperHistopath-defined risk groups in the Cancer Genome Atlas (TCGA) cohorts of patients with melanoma. **(A)** Kaplan-Meier Survival curves for patients in the high-risk group (blue) and low risk group (red) classified by stromal cells ratio derived from SuperHistopath and **(B)** Kaplan-Meier Survival curves for patients in the high-risk group (blue) and low risk group (red) classified by immune infiltrate based on lymphocytes cluster ratio derived from SuperHistopath. **(C, D)** show the SuperHistopath-based quantification of tumor phenotype in genetically-engineered mouse model of high-risk neuroblastoma. **(C)** Representative SuperHistopath-segmented whole-slide images (5x) and pie chart showing the Super-CNN quantified mean composition of the tumors arising in Th-MYCN (n=6) and Th-ALK^F1174L^/MYCN (n=7) mouse models of high-risk neuroblastoma. Note the marked difference of phenotype induced by the expression of the ALK^F1174L^ mutation characterized by **(D)** a significantly increased neuroblastoma differentiation neuroblasts and the total abrogation of the characteristic hemorrhagic phenotype of Th-MYCN tumors.

We first use SuperHistopath to quantify both the stroma-to-tumor ratio and the immune infiltrate, which have both shown to provide prognostic and predictive information in patient with solid tumors, including melanoma (20, 21, 23). The important role of immune hotspots has been established based on density analysis of single cell classification of lymphocytes in high-resolution images (61, 62). Here, we demonstrate in our melanoma dataset of 127 WSIs i) that a high stromal ratio as identified in low resolution WSIs is a predictor of poor prognosis (SuperHistopath: p = 0.028, Coxph-Regression [discretized by median]: HR = 2.1, p = 0.0315; **Figure 6A**) and ii) that clusters of lymphocytes hold predictive information in our melanoma dataset, with a high lymphocyte ratio being an indicator of favorable prognosis [SuperHistopath: p = 0.015, Coxph-Regression (discretized by median): HR = 0.4, p = 0.018; **Figure 6B**]. Pearson’s correlation showed no significant correlation between stromal ratio and clusters of lymphocytes ratio (r = -0.13, p = 0.13), and between absolute sizes of stroma and clusters of lymphocytes (r = 0.13, p=0.11). Taken together, our data, captured from low resolution (5x) WSIs, are consistent with those extracted from single-cell analysis in high-resolution WSIs (53).

#### Necrosis Quantification

We use the SuperHistopath to quantity tumor necrosis in our breast cancer and childhood neuroblastoma preclinical datasets. Tumor necrosis, defined as confluent cell death or large area of tissue damage hold predictive and prognostic information, both at diagnosis and after chemotherapy, in many solid tumors including breast cancer and childhood malignancies (24–26, 63, 64). While visible at 5x objective lens magnification, its quantification can often be a challenging task even for experienced pathologists. Here, we show that SuperHistopath can provide satisfactory quantification of necrosis in clinical breast cancer samples by distinguishing from stroma with high specificity (91.5%) and satisfactory precision (79.5%) and in the high-risk neuroblastoma mouse models with high precision and specificity (93.5% and 98.9% respectively).

#### Quantification of Neuroblastoma Differentiation

We used SuperHistopath to quantify the phenotype of MYCN-driven transgenic mouse models of high-risk stroma-poor neuroblastoma. We show that SuperHistopath can identify areas of differentiation, a critical feature for the stratification of children neuroblastoma, with both high precision and specificity (100% and 96.9% respectively). SuperHistopath also showed that expression of *ALK^F1174L^* mutation significantly shift the MYCN-driven phenotype from poorly-differentiated and hemorrhagic phenotype (Th-*MYCN*: 1.8 ± 1.3% differentiating area and 29.2 ± 6.7% hemorrhage, **Figure 6C**) into a differentiating phenotype also characterized by the almost complete abrogation of the hemorrhagic phenotype (Th-*ALK^F1174L^*/*MYCN*: 20.3 ± 3.1% differentiating area and 0.2 ± 0.1% hemorrhage, p=0.0003 and p=0.0008 respectively, **Figure 6D**) as previously demonstrated (51, 65).

## Discussion

In this study, we implemented SuperHistopath: a digital pathology pipeline for the classification of tumor regions and the mapping of tumor heterogeneity from low-resolution H&E-stained WSIs, which we demonstrated to be highly accurate in three types of cancer. Combining the application of the SLIC superpixels algorithm directly on low magnification WSIs (5x) with a CNN architecture for the classification of superpixels, contributes to SuperHistopath computational efficiency allowing for fast processing, whilst affording the quantification of robust and easily interpretable clinically-relevant markers.

Applying our computational approach on low-resolution images leads to markedly increased processing speed, for both the classification of new samples and network training. Here, we chose the (5x) magnification as a compromise between tumor structures visibility and computational cost. Specific metrics such as stroma-to-tumor ratio could potentially be derived from images at even lower magnifications (e.g. 1.25x) as recently shown (53). Digital histology images are conventionally processed at 40x (or 20x) magnification where cell morphology is most visible. At those resolutions, WSIs are large (representative size at 20x: 60000 x 60000 pixels), requiring of a lot of memory and images to be divided into patches (tiles) for processing. Under these conditions, the training of new networks for cell segmentation and classification typically requires days and the application to new WSI samples can take hours prior to code optimization. In contrast, the training of our neural network until acceptable convergence needed as little as ~30 min and application on new samples ~5 min (for both superpixel segmentation and classification) in our study. High-resolution images are essential when studying cell-to-cell interactions, however we show that the processing of low resolution images is appropriate for the extraction of specific global context features.

Furthermore, SuperHistopath combines the main advantages of regional classification and segmentation approaches. On one hand, classification approaches applied on smaller patches resulting from splitting WSIs allow the use of CNN for the robust classification of many categories necessary to capture intratumor heterogeneity (39), yet at the expense of higher risk of misclassification, especially close to regional boundaries where an image patch, regardless of its size, may contain multiple tumor components. Overlapping (sliding) window approaches can improve the issue, yet at an increased computational cost. On the other hand, segmentation approaches such as U-Net-like architectures can resolve the regional boundaries issue but appear to work better for few classes, typically two. SuperHistopath efficiently combines the use of a segmentation approach using superpixels to adhere to region boundaries with CNN classification to cover the rich tumor histological heterogeneity (here 6-8 region categories depending on the cancer type).

Our method also markedly simplifies and accelerates the process of preparing ground-truth (annotations) datasets as *i)* the use of superpixels alleviate the need for careful boundary delineation of the tumor components of interest (**Figure 1B**), a cumbersome and time-consuming process necessary for using U-Net-like architectures and ii) each annotated region contains large numbers of superpixels facilitating the collection of the large datasets traditionally required by deep learning methods.

The appropriate choice of superpixel size is crucial to warrant both accurate tissue segmentation and classification. Equation 1 ensured a uniform superpixel size for every whole-slide image regardless of their original size. The main considerations for choosing superpixels size (i.e. setting the constant *U*) is to ensure that they only contain a single tissue type, while being large enough to contain sufficient tissue information. In our study, we found that classification is not sensitive to small changes of *U*. However larger superpixels (*U* > 1750) did not adhere well to the tissue boundaries, whereas smaller superpixels (*U* < 1250) indeed led to a slight decrease in classification performance.

Many promising computational pathology-derived biomarkers ultimately fail to translate in the clinic due to their inherent complexity and the difficulty for pathologists to evaluate them in new datasets. In this proof-of-concept study, we showed that SuperHistopath can quantify well-understood features/markers already used, albeit only qualitatively or semi-quantitatively, by pathologists, including the stroma-to-tumor ratio, lymphocyte infiltration, tumor necrosis, and neuroblastoma differentiation. We also show that SuperHistopath-derived results corroborated those obtained from single-cell analysis on high-resolution samples (53). The computational efficiency of SuperHistopath, combined with the simple superpixels-enabled data collection, could facilitate its adoption in the clinic to accelerate pathologist workflow, could assist in intra-operative pathological diagnosis and should facilitate working with large datasets in clinical research.

Moving forward, we plan to expand the types of global context features extractable from SuperHistopath in more cancer types. We will also evaluate the accuracy of SuperHistopath on digitized frozen tissue sections to demonstrate its potential to assist in the rapid intra-operative pathological diagnostic. We will also update our previous framework (SuperCRF) which incorporates region classification information to improve cell classification (53) using SuperHistopath. Together both SuperHistopath and SuperCRF would provide invaluable tools to study spatial interactions across length scales to provide a deeper understanding of the cancer-immune-stroma interface, key to further unlock the potential of cancer immunotherapy (17).

In this proof-of-concept study, we applied our method to three cancer types with disparate histology without any changes (just retraining). While the approach could thus be virtually extended to any type of cancer, improvement could be made tailored to a specific global feature, cancer type or dataset and could include further exploring i) the use of SVM to combine the CNN-extracted features with handcrafted ones, ii) the use of other image color spaces which has been shown to improve classification in certain cases (66) and iii) alternative superpixel algorithms such as the efficient topology preserving segmentation (ETPS) algorithm (67). Additionally, further improvement of this proof-of-concept framework could be sought *via* experimentation with hyperparameter tuning, or the use of other custom and well-established architectures (59, 68). Since superpixels only capture small homogeneous areas, combination with other approaches such as classification of larger image patches with a deepCNN or U-net-like architectures might be more appropriate for the single purpose of segmenting some large and multi-component tumor structures, e.g. certain types of glands (16).

To conclude, our novel pipeline, SuperHistopath can accurately classify and map the complex tumor heterogeneity from low-resolution H&E-stained histology images. The resulting enhanced speed for both training and application (~5 min for classifying a WSI and as low as ~30 min for network training) and the efficient and simple collection of ground-truth datasets make SuperHistopath particularly attractive for research in rich datasets and would facilitate its adoption in the clinic to accelerate pathologist workflow in the quantification of predictive/prognosis markers derived from global features of interest.

## Data Availability Statement

The melanoma dataset comes the publicly available TCGA dataset. The neuroblastoma dataset is available from the corresponding authors upon reasonable request. The images from the triple-negative breast cancer dataset cannot be released yet due to ongoing clinical studies. The codes that support the findings of this study are available from the corresponding authors upon reasonable request.

## Ethics Statement

The breast cancer clinical dataset was generated from diagnostic H&E images provided anonymised to the researchers by the Serbian Institute of Oncology. The neuroblastoma preclinical dataset was built from H&E images collected during previous in vivo studies approved by The Institute of Cancer Research Animal Welfare and Ethical Review Body and performed in accordance with the UK Home Office Animals (Scientific Procedures) Act 1986. The melanoma clinical samples come from the publicly available TCGA dataset (**Table 1**).

## Author Contributions

Conception and design: KZ-P, IR, YJ, YY. Development of methodology: KZ-P Analysis and interpretation of data: KZ-P, RN, IR, YJ, YY. Administrative and/or material support: RN, DK, IR, YJ. Writing and review of the manuscript: KZ-P, IR, YJ, YY. IR, YJ, and YY are co-senior authors of this study. All authors contributed to the article and approved the submitted version.

## Funding

We acknowledge financial support by The Rosetrees Trust (KZ-P, M593). RN acknowledges funding from ISCIII (FIS) and FEDER (European Regional Development Fund) PI17/01558 and CB16/12/00484. YY acknowledges funding from Cancer Research UK Career Establishment Award (C45982/A21808), Breast Cancer Now (2015NovPR638), Children’s Cancer and Leukaemia Group (CCLGA201906), NIH U54 CA217376, and R01 CA185138, CDMRP Breast Cancer Research Program Award BC132057, CRUK Brain Tumor Awards (TARGET-GBM), European Commission ITN (H2020-MSCA-ITN-2019), Wellcome Trust (105104/Z/14/Z), and The Royal Marsden/ICR National Institute of Health Research Biomedical Research Centre. YJ is a Children with Cancer UK Research Fellow (2014/176). We thank Breast Cancer Now for funding IR as part of Programme Funding to the Breast Cancer Now Toby Robins Research Centre.

## Conflict of Interest

The authors declare that the research was conducted in the absence of any commercial or financial relationships that could be construed as a potential conflict of interest.
