# Supplementary material for: SuperHistopath: A Deep Learning Pipeline for Mapping Tumor Heterogeneity on Low-Resolution Whole-Slide Digital Histopathology Images
Source: Front Oncol. 2021 Jan 20;10:586292. doi: 10.3389/fonc.2020.586292 (PMC7855703; doi:10.3389/fonc.2020.586292)
Supplement: Supplementary file 1 [file Table_1.docx]

**Supplementary Table 1.** Confusion matrix of the classification of superpixels using our custom-made CNN in melanoma patients in 6 categories: tumor, stroma, normal epidermis, cluster of lymphocytes (Lym), fat and empty/white space (separate test set of 5 whole-slide images). Overall accuracy = 96.7%, average precision = 93.6%, average recall = 93.6%.

|  | **Tumor** | **Stroma** | **Epidermis** | **Lym** | **Fat** | **Empty space** |
| --- | --- | --- | --- | --- | --- | --- |
| **Tumor** | **5223** | 24 | 27 | 36 | 1 | 0 |
| **Stroma** | 24 | **937** | 33 | 2 | 1 | 0 |
| **Epidermis** | 95 | 0 | **473** | 0 | 0 | 0 |
| **Lym** | 8 | 0 | 1 | **812** | 0 | 0 |
| **Fat** | 0 | 20 | 2 | 0 | **5481** | 112 |
| **Empty space** | 0 | 1 | 0 | 0 | 83 | **695** |
