# Supplementary material for: SuperHistopath: A Deep Learning Pipeline for Mapping Tumor Heterogeneity on Low-Resolution Whole-Slide Digital Histopathology Images
Source: Front Oncol. 2021 Jan 20;10:586292. doi: 10.3389/fonc.2020.586292 (PMC7855703; doi:10.3389/fonc.2020.586292)
Supplement: Supplementary file 2 [file Table_2.docx]

**Supplementary table 2.** Confusion matrix of the classification of superpixels using our custom-made CNN in triple-negative breast cancer patients in 6 categories: tumor, necrosis, cluster of lymphocytes (Lym), stroma, fat and lumen/empty space (separate test set of 5 whole-slide images). Overall accuracy = 91.7%, average precision = 92.5%, average recall = 91.8%.

|  | **Tumor** | **Necrosis** | **Lym** | **Stroma** | **Fat** | **Empty space** |
| --- | --- | --- | --- | --- | --- | --- |
| **Tumor** | **1828** | 35 | 3 | 34 | 0 | 0 |
| **Necrosis** | 89 | **1350** | 10 | 369 | 0 | 0 |
| **Lym** | 5 | 0 | **701** | 15 | 0 | 0 |
| **Stroma** | 60 | 102 | 23 | **3833** | 0 | 1 |
| **Fat** | 0 | 2 | 4 | 18 | **538** | 5 |
| **Empty space** | 0 | 1 | 0 | 6 | 71 | **1246** |
