# Supplementary material for: SuperHistopath: A Deep Learning Pipeline for Mapping Tumor Heterogeneity on Low-Resolution Whole-Slide Digital Histopathology Images
Source: Front Oncol. 2021 Jan 20;10:586292. doi: 10.3389/fonc.2020.586292 (PMC7855703; doi:10.3389/fonc.2020.586292)
Supplement: Supplementary file 3 [file Table_3.docx]

**Supplementary table 3.** Confusion matrix of the classification of superpixels using our custom-made CNN in the Th-*MYCN* and Th*-ALK^F1174L^/MYCN* mouse models in 8 categories: region of undifferentiated neuroblasts, necrosis, cluster of lymphocytes (Lym), hemorrhage (blood), empty/white space, muscle tissue and kidney (separate test set of 16 whole-slide images). Overall accuracy = 96.8%, average precision = 97.1%, average recall = 97.2%.

|  | **Undiffe-**  **rentiated**  **region** | **Necrosis** | **Lym** | **Differenti-**  **ation** | **Blood** | **Empty space** | **Muscle** | **Kidney** |
| --- | --- | --- | --- | --- | --- | --- | --- | --- |
| **Undiffe-rentiated region** | **1419** | 1 | 0 | 1 | 0 | 0 | 0 | 0 |
| **Necrosis** | 33 | **1566** | 2 | 76 | 60 | 1 | 3 | 15 |
| **Lym** | 46 | 4 | **1114** | 0 | 0 | 0 | 0 | 0 |
| **Differenti-ation** | 18 | 0 | 0 | **1240** | 1 | 0 | 0 | 2 |
| **Blood** | 0 | 5 | 0 | 7 | **1330** | 0 | 2 | 0 |
| **Empty space** | 0 | 4 | 0 | 0 | 0 | **545** | 15 | 3 |
| **Muscle** | 0 | 0 | 0 | 9 | 1 | 0 | **1166** | 3 |
| **Kidney** | 0 | 1 | 0 | 0 | 0 | 0 | 0 | **1175** |
